# Prevalence and Associated Factors of Benzodiazepine Use in Patients Attending a Community Mental Health Team in Scotland: A Cross-Sectional Survey

**DOI:** 10.2174/0117450179375369250623061653

**Published:** 2025-06-27

**Authors:** Felix Kauye, Amanthi Wijesundara, Dalitso Mwandumba

**Affiliations:** 1 Consultant Psychiatrist, Helensburgh CMHT, Helensburgh, Scotland, UK; 2 Medical Student, University of Glasgow, Glasgow, Scotland, UK; 3 Resident Doctor, Helensburgh CMHT, Helensburgh, Scotland, UK; 4 Gartnavel Royal Hospital, Glasgow, Scotland, UK

**Keywords:** Benzodiazepine, Community Mental Health Team, Secondary Care, Prevalence, Associated Factors, Long-Term, Scotland

## Abstract

**Introduction:**

We aimed to assess the point prevalence and associated factors of Benzodiazepine (BZD) use in patients attending a Community Mental Health Team (CMHT) in Scotland.

**Methods:**

This cross-sectional survey included 412 patients from the outpatient medical caseload over a three-month period in 2021. Patient records were reviewed to identify whether they were prescribed BZDs. The analysis aimed to determine the point prevalence rate of BZD use and compare characteristics between patients on and not on BZDs.

**Results:**

The point prevalence rate was 16%. There were no significant sex differences (p=0.10) between patients on and not on BZDs. However, statistically significant differences were observed in relation to age (p=0.003), primary diagnosis (p=0.03), and the number of psychotropic medications (p= <0.001). Only the number of psychotropic medications varied significantly between long and short-term BZD use (p=0.005). Those on long-term BZD use of one year or longer had a higher number of psychotropic medications.

**Discussion:**

Monitoring and comparing the prevalence rates of BZD prescription by CMHTs is essential for reducing adverse effects associated with BZDs. Such concerns can influence clinical practice and may sometimes lead to conflicts between secondary and primary care clinicians. This study involved only one CMHT in Scotland and, therefore, may not be fully representative of all CMHTs across the country.

**Conclusion:**

A high number of psychotropic medications emerged as the only statistically significant factor associated with long-term BZD use. Consequently, diligent monitoring of BZD use is warranted in patients on a high number of psychotropic medications.

## 1 INTRODUCTION

There is global concern regarding the long-term use of benzodiazepines (BZDs) and Z-drugs [1]. BZDs are the most prescribed anxiolytic medication [2]. BZDs work significantly more rapidly for anxiety than antidepressants, are used for various indications in different healthcare settings, and are not limited to mental health services only. Due to their rapid onset and immediate symptom relief, BZDs are commonly used to treat conditions, such as sleep disorders, anxiety, spasticity resulting from central nervous system (CNS) pathology, muscle tension, and epilepsy [3]. Alprazolam has had Food and Drug Administration (FDA) approval for generalized anxiety disorder (GAD) since DSM-3, and BZDs have demonstrated efficacy for both somatic and psychic symptoms associated with GAD [4]. Furthermore, BZDs are widely used in different countries for agitation and rapid tranquilisation [5]. Oral BZDs are used as first-line treatment for mild cases of agitation and are often used in conjunction with antipsychotics; however, they are less effective than antipsychotics in severe cases of agitation [4].

Adverse effects associated with BZDs include sedation, falls, and cognitive impairment [6]. Sedation can be problematic, and due to decreased alertness, it increases the risk of car accidents and falls [6]. Rates of deaths caused by BZD overdose in the USA increased between the years 2000 and 2017 before decreasing from 2018 to 2019 [7]. The 2018 to 2019 period coincided with decreased rates of overdoses involving benzodiazepines and natural and semi-synthetic opioids [7]. The risks are more pronounced when BZDs are combined with alcohol, opioids, or other medications that affect the CNS [8]. There is limited evidence of a link between BZD use and dementia risk [9], and doubt persists in the biomedical community regarding the relatively new safety concerns (dementia, infections, pancreatitis, and cancer) regarding BZDs and Z-drugs [10]. One of the primary concerns regarding the use of BZDs is the risk of dependency, and guidelines advocate for short-term or intermittent use [11]. Long-term use of BZDs increases the risk of dependence [12].

The definitions of short-term and long-term BZD use can vary [13, 14]. According to Fluyau *et al.* [15], it can take between one to six months for tolerance, dependence, and the risk of withdrawal to develop. A review conducted by Brett and Murnion [16] reported that tolerance and dependence can develop if BZDs are used for more than four weeks. In the UK, short-term use means using BZDs for less than four weeks, whereas in France, short-term use is defined as taking BZDs for less than three months [14]. The French recommendations on duration of short-term BZD use distinguish between hypnotic (4 weeks) and anxiolytic BZDs (12 weeks) [17].

Additionally, studies use different descriptions of long-term BZD use, and this varies from four weeks to many years [12, 18]. For this study, we defined long-term BZD use as patients who have been on BZDs for one year or more. In addition to determining the overall point prevalence of BZD use, we compared the characteristics of patients prescribed BZDs with those not on BZDs, as well as patients on long-term BZD use with those on short-term use.

One study on BZD prescriptions in the USA used data from the National Ambulatory Medical Care Survey and analysed prescription patterns between 2003 and 2015 [8]. The study examined 20,884 and 24,273 visits in 2003 and 2015, respectively. The prescription rates of BZDs by psychiatrists in the USA did not change significantly between 2003 and 2015 (29.6% and 30.2%, respectively). This study also observed other healthcare providers, including general practitioners (GPs) and general physicians, and found that the prescriptions of BZDs had doubled from 3.8% in 2003 to 7.4% in 2015. However, the prescription rates by psychiatrists remained constant at approximately 30%.

Most studies that have examined the prescribing of BZDs and factors associated with BZD use have been conducted in the general population. These studies have identified factors such as female sex and older age as being associated with BZD use [19, 20]. Long-term BZD use has been linked to increasing age and prescribing-related factors, including the initiation of multiple BZDs and the concurrent use of other medications [21]. Additionally, individuals with a history of BZD prescriptions are four times more likely to misuse them, with the most common source of misused BZDs being family or friends [22].

Few studies have examined BZD use among patients attending mental health services [23], and most existing research has focused on high-risk groups, such as individuals with substance use disorders or depression [24]. A retrospective study from the USA involving patients at community mental health services across fourteen sites found that 19.9% of all patients were prescribed at least one BZD at the time of chart review [25]. Among those prescribed a BZD, 35.1% had a co-occurring Substance Use Disorder (SUD). The majority of BZD recipients were female (61.7%), white (73.4%), and aged 55 or older (42.9%). BZD prescribing was significantly associated with age, sex, race (all p < .01), and the presence of an anxiety disorder (p < .05).

The present study aimed to assess BZD prescribing and use among patients attending a community mental health service in Helensburgh, Scotland. We also compared the characteristics of patients prescribed BZDs with those who were not and examined the prevalence and associated factors of long-term BZD use.

## 2 METHODS

### 2.1 Type of Survey

A cross-sectional survey was conducted to examine BZD prescribing and use in a cohort of patients on the caseload of the Helensburgh Community Mental Health Team (CMHT). The study included all 412 patients registered for outpatient clinics between May and August 2021.

According to Naing *et al.* [26], for an expected prevalence of 30% of BZD use in psychiatrist-run outpatient clinics [8], the required sample size is 359 to achieve a margin of error, or absolute precision, of 5% in estimating the prevalence with 95% confidence, considering a potential loss/attrition of 10%. With this sample size, the anticipated 95% confidence interval is 20% to 40%. Our sample size exceeded 359 patients.

### 2.2 Study Area

The Helensburgh and Lomond areas, part of the Argyll and Bute Council, have a catchment population of 25,715 [27]. The region is served by five general practitioner (GP) practices, and between October 2021 and September 2022, the Helensburgh Community Mental Health Team (CMHT) received 494 referrals [28]. Geographically, the Helensburgh CMHT is located within the NHS Highlands region; however, administratively, it falls under NHS Greater Glasgow and Clyde. At the time of the survey, the CMHT had one consultant psychiatrist responsible for both inpatient and outpatient care, conducting three outpatient clinic sessions per week.

### 2.3 Data Collection and Analysis

The list of 412 patients included in the study was extracted from the Electronic Medical Information System (EMIS) and comprised all patients registered with the Helensburgh CMHT outpatient clinics. Data on BZD prescriptions were obtained from the Clinical Portal, where GP prescriptions are recorded. After compiling the list of patients prescribed BZDs using data from the Clinical Portal, the duration of BZD use was extracted also from their EMIS records. All data were entered into an Excel spreadsheet, which also contained other variables that were not used in this analysis.

This analysis aimed to determine the point prevalence rate of BZD use and compare the characteristics of different patient groups. The characteristics of the following groups were compared:

Patients on BZDs vs. those not on BZDs

Patients on short-term BZD use vs. those on long-term BZD use

Continuous factors were approximately normally distributed and were compared between groups using the unpaired t-test. The Chi-square test was used to compare categorical variables between groups.

Community Mental Health Teams (CMHTs) are secondary care providers of mental health services and receive referrals from primary care. Once a referral is accepted, the patient is registered with the CMHT. This study included all 412 patients who were open to the Helensburgh CMHT and actively attending outpatient clinics. No patients were excluded from the study.

## 3 RESULTS

The overall sample comprised records of 412 patients with no missing data. The sample was predominantly female (N = 241, 58.5%), and 26.2% (N = 108) were aged 55 years or older. Only two patients (0.5%) were aged over 70 years, and there were no patients below 18 years of age. The most common primary diagnosis was recurrent depressive disorder (N = 84, 20.4%), followed by psychotic disorders (N = 57, 13.8%), Emotionally Unstable Personality Disorder/Borderline Personality Disorder (EUPD/BPD) (N = 56, 13.6%), and bipolar disorder (N = 36, 8.7%). Generalized Anxiety Disorder (GAD) accounted for only 2.9% (N = 12) of the total sample.

### 3.1 Benzodiazepines

The first analysis examined BZD use and compared the characteristics of patients on BZDs with those not on BZDs. Of the 412 patients included in the study, 65 (16%) were prescribed BZDs.

A summary of the analysis results is presented in Table **1**. Patient characteristics for both groups, those on BZDs and those not on BZDs, are provided. Categorical variables are summarized by the number and percentage within each group, while continuous variables are reported as mean and standard deviation. P-values indicating the significance of differences between groups are shown in the final column.

The analysis revealed no significant sex differences between patients on BZDs and those not on BZDs. However, statistically significant differences were found in age, primary diagnosis, and the number of psychiatric medications.

Patients who were on BZDs were typically older, with a mean age of almost 48 years, compared to a mean of almost 42 years for those not on BZDs. The primary diagnosis indicated that patients on BZDs were more likely to have a diagnosis of EUPD/BPD than those not on BZDs but less likely to have a recurrent depressive disorder diagnosis. Additionally, the mean number of psychotropic medications was higher among patients on BZDs (3.3) compared to those not on BZDs (1.7).

### 3.2 Duration of Benzodiazepines

The next analyses examined the differences between patients on short-term BZDs (< one year) and those on long-term BZDs (one year or longer).

There were 65 patients on BZDs, and almost half of these (32 patients - 49%) were on short-term BZD use, while just over half (33 patients - 51%) were on long-term BZD use and taking them for one year or longer. The ones on long-term BZD use were 8% of the total sample.

Comparisons between these two groups are summarized in Table **2**.

Only the number of psychotropic medications differed significantly between long-term and short-term BZD users. Patients on long-term BZD use had a higher average number of psychotropic medications, with a mean of 3.7 compared to 2.9 in the short-term group.

There was some indication that the long-term BZD group included a higher proportion of males; however, this difference was not statistically significant. Age, primary diagnosis, and frequency of use did not significantly differ between short- and long-term BZD users.

Among patients on long-term BZD use (over one year), 45% were taking BZDs daily, while the remaining 55% used them intermittently or as needed (PRN). This corresponds to 23.1% of all patients on BZDs being on long-term daily use.

## 4 DISCUSSION

The point prevalence of BZD use in this study was 16%, with 8% of the total sample on long-term use (one year or longer). Significant differences between patients on BZDs and those not on BZDs were found in age, primary diagnosis, and the number of psychotropic medications. When comparing long-term and short-term BZD users, only the number of psychotropic medications differed significantly; other factors, including age, showed no significant differences.

The prevalence rate of BZD use varies across different populations and care settings. In the general population, prevalence rates ranging from 3.2% to 18.6% have been reported, with higher rates among individuals with psychiatric disorders, ranging from 9.2% to 41.9% [29]. From 2017 to 2018, 1.4 million (3%) adults in England received and dispensed one or more BZD prescriptions [30]. In the USA, approximately 5.2% of adults used benzodiazepines in 2008 [19]. A retrospective cohort study of adult patients attending primary care settings in a large Ohio healthcare system found that BZDs were prescribed in 3.2% of visits [31]. Our prevalence rate of 16% is comparable to and occasionally lower than, rates found in other studies in similar secondary care settings. A South African study [32], which examined records of patients aged 18 years and older attending community psychiatry clinics over a six-month period in 2019, found that approximately 25% of patients were prescribed BZDs. An audit of BZD prescribing in patients attending a community mental health team in North Shields, England, over one year in 2005 reported a rate of 24% [33]. In the USA study [8], the prevalence of BZD prescriptions by psychiatrists was around 30% and remained unchanged between 2003 and 2015.

Different factors have been identified to be associated with BZD use. Studies in general populations have shown BZD use to be higher in females than in males [19, 34], and that age is the most consistent predictor of BZD use [24]. Although our study did not show significant sex differences in BZD use, the association of higher prevalence rates in females than males has been shown in different countries, such as Spain [24] and Pakistan [35]. Additionally, BZD use was higher in males than females in patients attending community psychiatric clinics in South Africa [32]. Unlike our finding of the association of BZD use with older patients, a Spanish study [24] found that age was not significantly associated with BZD use. The study in Pakistan [35] also found increasing age to be associated with an increased likelihood of BZD use in psychiatric outpatient clinics.

In our study, patients on BZDs were more likely to have a diagnosis of EUPD/BPD than those not on BZDs but less likely to have a diagnosis of recurrent depressive disorder. In the Spanish study [24], BZDs were prescribed to 87% and 67% of patients diagnosed with a general or severe psychiatric disorder, respectively. Furthermore, 81% of patients diagnosed with an affective disorder were prescribed BZDs, while 64% of patients diagnosed with non-affective disorders took BZDs. Affective disorders included depressive, anxiety-depressive, anxiety, schizoaffective, bipolar, and personality disorders. Non-affective disorders included schizophrenia, psychosis, and delusion disorder. Wang *et al.* [29] found that the prevalence of BZD use among patients with major depressive disorder was 42.9%. In the South African study [32], the most common psychiatric diagnoses among patients on BZDs were bipolar and psychotic disorders, with the majority having no comorbid medical illnesses or substance use. An epidemiological study in the USA [36] reported that Emotionally Unstable Personality Disorder/Borderline Personality Disorder (EUPD/BPD) is rarely diagnosed alone, with high lifetime prevalence rates of anxiety disorders (84.8%), mood disorders (82.7%), substance use disorders (SUD; 78.2%), and eating disorders (ED; 33.7%). Comorbidity may explain why patients diagnosed with EUPD/BPD are often prescribed BZDs. In our study, the overall proportion of patients diagnosed with Generalized Anxiety Disorder (GAD) (2.9%) was lower compared to those with EUPD/BPD (13.6%), which explains why more patients on BZDs had a primary diagnosis of EUPD/BPD than GAD. The proportion of patients with a diagnosis of GAD who were prescribed BZDs (50%) was higher than that of patients with a diagnosis of EUPD/BPD prescribed BZDs (23.2%). Patients diagnosed with GAD were the most likely to be prescribed BZDs.

Our rate of long-term BZD use, at 8% of the total sample, was slightly lower than the 10% to 19% range reported in some studies [17, 18, 37]. Studies that have assessed long-term BZD use at various cut-off points [18] found that prevalence rates were highest at the lowest cut-off and lowest at the highest cut-off. The South African study [32], which defined long-term use as exceeding 180 days, found that nearly 90% of patients on BZDs were prescribed them long-term. In our study, using a one-year duration as the cut-off for long-term use, 51% of patients on BZDs had been taking them for one year or longer, including those using them intermittently or as needed (PRN). Less than half of the long-term users (23.1%) were prescribed BZDs on a regular daily basis. Although BZD use has declined over recent decades in most countries, long-term BZD use remains common in the 2000s, affecting 30 to 40% of all patients on BZDs [18].

The association between long-term BZD use and a higher number of psychotropic medications is consistent with findings from other studies [21]. Concurrent prescriptions of multiple BZDs [38] have also been linked to long-term use. Although our results did not show an association between age and long-term BZD use, strong evidence supports increasing age as a significant factor in long-term BZD use [39-41]. Findings on the influence of sex [42, 43] and socioeconomic status [40, 43] on long-term BZD use are mixed. Other factors studied in relation to long-term use include treatment-related characteristics [44] and prescriber-related factors [21]. The use of short-acting, high-potency BZDs such as alprazolam, lorazepam, or oxazepam has been identified as a significant predictor of long-term use [44]. Additionally, prescriber characteristics, including psychiatrist involvement, have been associated with higher rates of long-term BZD prescribing [21, 45].

One of the main concerns with long-term BZD use is the potential risk of dependency [16]. Guidelines for BZD use recommend that if BZDs are needed, they should be prescribed for short-term use or taken intermittently every few days [11]. Prescribers must also weigh the risks of untreated illnesses [46]. According to the recommendations from the representatives of the Psychopharmacology Special Interest Group of the Royal College of Psychiatrists of the UK and the British Association for Psychopharmacology, if there is no history of drug dependence and positive indicative lifestyle factors are present, a conscious decision to continue BZD treatment may be more reasonable than alternatives. However, the patient should periodically attempt to slowly reduce the dosage at regular intervals and try to stop altogether when or if possible [11].

In England, from 2015 16 to 2017 18, the number of people with a dispensed prescription for BZDs decreased by 5.1% (from 1.42 million) and Z-drugs by 5.0% (from 1.04 million) [47]. Prescribing and long-term prescribing estimates showed differences by region. General practitioners in the north and east of England were more likely to prescribe antidepressants, opioids, and gabapentinoids, whereas BZDs and Z-drugs were prescribed more frequently in the southwest, southeast, and east. The lower rate of BZD and Z-drug prescribing in much of northern England is believed to be linked to several local non-governmental support services for patients experiencing long-term harm from BZDs in these regions. The efforts and legacy of these services are thought to have increased awareness within the Clinical Commissioning Groups (CCGs) [47].

## LIMITATIONS

This cross-sectional survey utilized an existing dataset and did not collect new data; therefore, it was limited to the variables already available. As cross-sectional studies capture data at a single point in time, they cannot establish long-term trends. Our analysis considered only the primary diagnosis and did not account for comorbid conditions, such as substance use disorders. Additionally, the study involved only one CMHT in Scotland, which may limit the generalizability of the findings to other CMHTs across the region. The relatively small sample sizes in some subgroups may have reduced the ability to detect statistically significant differences in certain analyses.

## CONCLUSION

Although the rates of BZD use in our study are lower than those reported in some similar settings, it remains important to monitor BZD prescribing and consider alternatives where possible. Patients on BZDs were more likely to have a diagnosis of EUPD/BPD, while those with a diagnosis of GAD were more likely to be prescribed BZDs. Therefore, assessing comorbid anxiety disorders in patients with EUPD/BPD should be an integral part of reviewing BZD use in this population. More than half of the patients on BZDs were long-term users, although less than a quarter were on regular long-term BZD treatment. The number of psychotropic medications was a significant indicator, with patients taking a higher number more likely to use BZDs long-term. This underscores the need for diligent monitoring and review of BZD prescribing in patients receiving multiple psychotropic medications. Prescribers must also carefully balance the risks of untreated illness against the risks of BZD use; in some cases, continuing BZD treatment may be the most reasonable option. Lastly, coordinated, individualised care plans for patients on long-term BZD use are strongly recommended.

## Figures and Tables

**Table 1:** Comparison of the characteristics of patients on and not on BZDs.

Factor	Category	No Benzos (n=347)	Benzodiazepines (n=65)	P-value
Sex	Female	197 (57%)	44 (68%)	0.10
	Male	150 (43%)	21 (32%)	-
Age	-	41.8 ± 15.1	47.7 ± 12.7	**0.003**
Primary	None	17 (5%)	0 (0%)	**0.03**
Diagnosis	Attention Deficit Hyperactivity Disorder (ADHD)	19 (5%)	1 (2%)	-
	Autistic Spectrum Disorder (ASD)	13 (4%)	0 (0%)	-
	Bipolar disorder	31 (9%)	5 (8%)	-
	EUPD/BPD	43 (12%)	13 (20%)	-
	GAD	8 (2%)	4 (6%)	-
	Mixed Anxiety Depressive Disorder	17 (5%)	7 (11%)	-
	Other Psychotic disorders	13 (4%)	2 (8%)	-
	Post Traumatic Stress Disorder (PTSD)	15 (4%)	5 (8%)	-
	Recurrent Depressive Disorder	77 (22%)	7 (11%)	-
	Schizophrenia	34 (10%)	8 (12%)	-
	Other	60 (17%)	13 (20%)	-
Number of psychotropic medications	-	1.7 ± 1.1	3.3 ± 1.2	**<0.001**

Summary statistics are: mean ± standard deviation or number (percentage).

**Table 2:** Comparison between the characteristics of long-term and short-term BZD use.

Factor	Category	< 1 year (n=32)	≥ 1 year (n=33)	P-value
Sex	Female	25 (78%)	19 (58%)	0.08
	Male	7 (22%)	14 (42%)	-
Age	-	46.3 ± 13.9	49.1 ± 11.5	0.37
Primary	Bipolar disorder	2 (6%)	3 (9%)	0.13
Diagnosis	EUPD/BPD	6 (19%)	7 (21%)	-
	Mixed Anxiety / Depressive disorder	5 (16%)	2 (6%)	-
	PTSD	4 (13%)	1 (3%)	-
	Recurrent Depressive Disorder	2 (6%)	5 (15%)	-
	Schizophrenia	1 (3%)	7 (21%)	-
	Other	12 (38%)	8 (24%)	-
Number of psychotropic medications	-	2.9 ± 1.3	3.7 ± 1.1	**0.005**
Frequency use	Pro Re Nata (PRN)	15 (47%)	18 (55%)	0.54
-	Regular	17 (53%)	15 (45%)	-

Summary statistics are: mean ± standard deviation or number (percentage).

## Data Availability

The authors may deposit their datasets openly to Zenodo Repository, in addition to their own or their institutional archives. For more details please visit https://clinical-practice-and-epidemiology-in-mental-health.com/.

## LIST OF ABBREVIATIONS

ADHD: = Attention Deficit Hyperactivity Disorder
ASD: = Autism Spectrum Disorder
BPD: = Borderline Personality Disorder
BZD: = Benzodiazepine
CCG: = Clinical Commissioning Group
CMHT: = Community Mental Health Team
CNS: = Central Nervous System
ED: = Eating Disorder
EMIS: = Electronic Medical Information System
EUPD: = Emotionally Unstable Personality Disorder
FDA: = Food and Drug Administration
GAD: = Generalised Anxiety Disorder
GP: = General Practitioner
NHS: = National Health Service
PRN: = Pro Re Nata
PTSD: = Post Traumatic Stress Disorder
SUD: = Substance Use Disorder
USA: = United States of America
UK: = United Kingdom

## STANDARD OF REPORTING:

STROBE guidelines were followed.
